# The Creativity-Verification Cycle in Psychological Science: New Methods to Combat Old Idols

**DOI:** 10.1177/1745691618771357

**Published:** 2018-07-02

**Authors:** Eric-Jan Wagenmakers, Gilles Dutilh, Alexandra Sarafoglou

**Affiliations:** 1 Department of Psychology, University of Amsterdam; 2 Department of Economic Psychology, University of Basel

**Keywords:** preregistration, empirical cycle, philosophy of science

## Abstract

Over the years, researchers in psychological science have documented and investigated a host of powerful cognitive fallacies, including hindsight bias and confirmation bias. Researchers themselves may not be immune to these fallacies and may unwittingly adjust their statistical analysis to produce an outcome that is more pleasant or better in line with prior expectations. To shield researchers from the impact of cognitive fallacies, several methodologists are now advocating preregistration—that is, the creation of a detailed analysis plan before data collection or data analysis. One may argue, however, that preregistration is out of touch with academic reality, hampering creativity and impeding scientific progress. We provide a historical overview to show that the interplay between creativity and verification has shaped theories of scientific inquiry throughout the centuries; in the currently dominant theory, creativity and verification operate in succession and enhance one another’s effectiveness. From this perspective, the use of preregistration to safeguard the verification stage will help rather than hinder the generation of fruitful new ideas.

Psychological science appears to be in the midst of a transformation, one in which researchers from several subdisciplines have started to publicly emphasize methodological rigor and replicability more than they did before (e.g., Lilienfeld, 2017; Lindsay, 2015; Pashler & Wagenmakers, 2012; Spellman, Gilbert, & Corker, 2018). A prominent example of the new appreciation for rigor is the widespread adoption of *preregistration*, where researchers detail their substantive hypotheses and statistical analysis plan in advance of data collection or data inspection (e.g., Nosek, Ebersole, DeHaven, & Mellor, in press; Nosek & Lindsay, 2018; van ‘t Veer & Giner-Sorolla, 2016). The goal of such preregistration is to allow a sharp distinction between what was preplanned and what is post hoc (e.g., Chambers, 2017; de Groot, 1956/2014, 1969; Peirce, 1883).

To appreciate its steep rise to prominence, consider that only 7 years ago, few researchers in psychology had even heard of preregistration. Now, preregistration

- is a key component of the Registered Replication Reports, an article type for multilab replication studies recently initiated at *Perspectives on Psychological Science* and now published in *Advances in Methods and Practices in Psychological Science*;
- is a key component of Chambers’s Registered Reports, an outcome-independent publication format that is currently offered by 88 journals, including *Nature Human Behavior* (for details, see https://cos.io/rr/);
- is part of the Transparency and Openness Promotion guidelines (Nosek et al., 2015) that more than 5,000 journals and societies have under consideration (for more information, see https://cos.io/our-services/top-guidelines/);^[Fn fn2-1745691618771357]^
- is facilitated by online resources such as the Open Science Framework and aspredicted.org; and
- has been adopted in the form of a preregistration badge by the journal *Psychological Science*. In a recent editorial for *Psychological Science*, Editor Steve Lindsay stated that “Personally, I aim never again to submit for publication a report of a study that was not preregistered” (Lindsay, 2015, p. 1827).

The prospect of psychology as a preregistration-dominant discipline does not appeal to everybody. One worry is that preregistration will curtail creativity—essential for any scientific endeavor—and consequently stifle scientific progress. By tightening the methodological screws and increasing the demands on verification, one may inadvertently punish exploration and push researchers to stay within well-trodden paths (for a discussion, see Vazire, 2018). Will psychological science be less interesting when preregistration becomes the norm?

In this article, we provide a historical perspective on the interplay between creativity and verification. We hope to show that, within the common models of scientific inquiry, the processes of creativity and verification are relevant in separate stages, and increasing the quality of one process will increase the quality of the other.

## Historical Perspective

### Bacon’s idols

**Fig. 1.**
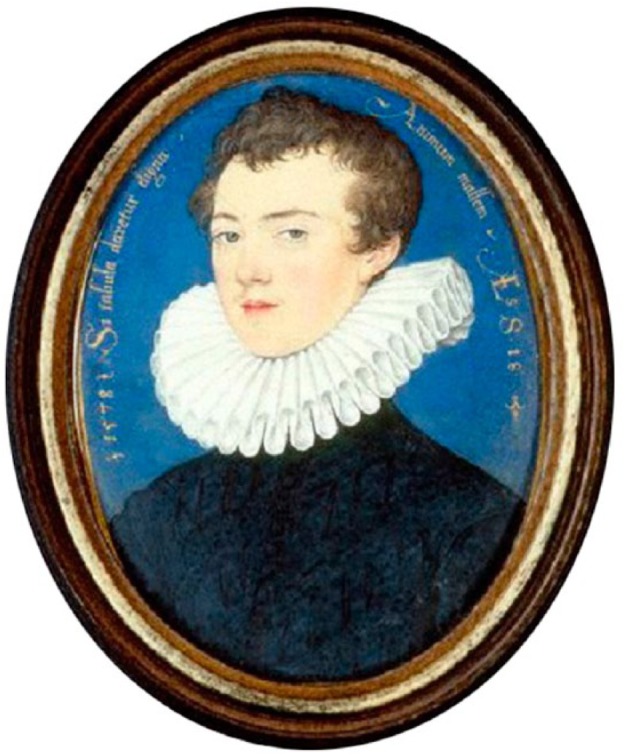
Sir Francis Bacon (1561–1626) depicted at 18 years of age. The inscription around his head reads “Si tabula daretur digna animum mallem” (“If one could but paint his mind). Painting by Nicholas Hilliard from the collection of the National Portrait Gallery, London. Public-domain image available at https://en.wikipedia.org/wiki/Francis_Bacon#/media/File:18-year_old_Francis_Bacon.jpg

Sir Francis Bacon (1561–1626; Fig. 1) is often considered the father of the modern scientific method. In several highly influential books, Bacon outlined a systematic and empirical approach to scientific learning, emphasizing the importance for researchers to question existing dogma and to overcome the biases—whether innate, social, or individual—that prey on the human mind. In an even-handed review of Francis Bacon’s work, Whewell (1840) states that “if we must select some one philosopher as the Hero of the revolution in scientific method, beyond all doubt Francis Bacon must occupy the place of honour” (p. 392).

Closer inspection, however, reveals that opinion on Bacon’s work is divided. For instance, Jevons (1877/1913) argues that “Francis Bacon, although he correctly insisted upon constant reference to experience, had no correct notions as to the logical method by which from particular facts we educe laws of nature” (p. ix), and Peirce (1877) claims that

superior as Lord Bacon’s conception is to earlier notions, a modern reader who is not in awe of his grandiloquence is chiefly struck by the inadequacy of his view of scientific procedure. That we have only to make some crude experiments, to draw up briefs of the results in certain blank forms, to go through these by rule, checking off everything disproved and setting down the alternatives, and that thus in a few years physical science would be finished up—what an idea! (p. 2)

Whewell (1840) identifies the same weakness:

Another mistake, which could not fail to render it unlikely that Bacon should really exemplify his precepts by any actual advance in science, was, that he did not justly appreciate the sagacity, the inventive genius, which all discovery requires. . . . he speaks of proceeding by *due rejections*; and appears to imagine that when we have obtained a collection of facts, if we go on successively rejecting what is false, we shall at last find that we have, left in our hands, that scientific truth which we seek. I need not observe how far this view is removed from the real state of the case. (pp. 402–403)

The most scathing critique of Francis Bacon and his work came from Justus von Liebig, a famous German chemist. Baron von Liebig examined Bacon’s method in painful detail, noted the same deficiency as Whewell, and concluded, “An experiment not preceded by a theory—that is, by an idea—stands in the same relation to physical investigation as a child’s rattle to music” (von Liebig, 1863, p. 263).^1^

Nevertheless, Francis Bacon provided a series of valuable insights concerning the scientific process (e.g., the emphasis on systematic experimentation, the dangers of jumping to conclusions, the questionable status of many scientific insights from antiquity, the importance of skepticism); the insight that is most relevant here is that researchers need to shield themselves against fallacies that beset all human minds:

As for the detection of False Appearances or Idols, Idols are the deepest fallacies of the human mind. For they do not deceive in particulars, as the others do, by clouding and snaring the judgment; but by a corrupt and ill-ordered predisposition of mind, which as it were perverts and infects all the anticipations of the intellect. For the mind of man (dimmed and clouded as it is by the covering of the body), far from being a smooth, clear, and equal glass (wherein the beams of things reflect according to their true incidence), is rather like an enchanted glass, full of superstition and imposture. Now idols are imposed upon the mind, either by the nature of man in general; or by the individual nature of each man; or by words, or nature communicative. The first of these I call Idols of the *Tribe.* . . .

As an example of the Idols of the Tribe, take this. The nature of the human mind is more affected by affirmatives and actives than by negatives and privatives; whereas by right it should be indifferently disposed towards both. But now a few times hitting or presence produces a much stronger impression on the mind than many times failing or absence: a thing which is as the root of all vain superstition and credulity. And therefore it was well answered by one who when the table was shown to him hanging in a temple of such as had paid their vows upon escape from shipwreck, and he was pressed to say whether he did not now acknowledge the power of Neptune, “Yea,” asked he in return, “but where are they painted that were drowned after paying their vows?” And so it is in similar superstitions, as astrology, dreams, omens, and the like. (Bacon, 1605/1858, p. 432)

Evidently inspired by Cicero,^2^ the “temple of Neptune” passage provides an example of survival bias, which in its modern academic manifestation is known as *publication bias*: The literature contains mostly articles that report statistically significant results. These are the visible studies that were “saved from academic shipwreck,” whereas those that did not achieve statistical significance tend to hide in the file drawer.

Francis Bacon’s concern with the fallacies of the human mind should not surprise the modern-day scientist: A quick search of the literature reveals more than 180 pervasive cognitive biases that have been documented in psychology and behavioral economics (e.g., see https://en.wikipedia.org/wiki/List_of_cognitive_biases). These biases include hindsight bias (e.g., Fischhoff, 1975), confirmation bias (e.g., Nickerson, 1998), and the bias blind spot—that is, the tendency to falsely believe that one’s own judgment is unaffected by bias (Pronin, Lin, & Ross, 2002).

**Fig. 2.**
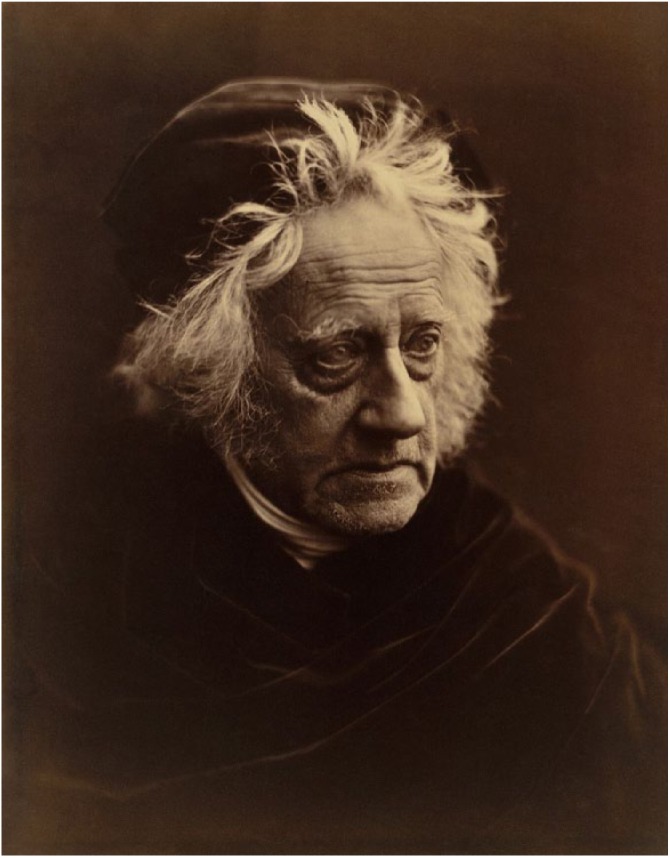
Sir John Herschel (1792–1871), as photographed in 1867 by Julia Margaret Cameron. Public-domain image available at https://en.wikipedia.org/wiki/John_Herschel#/media/File:Julia_Margaret_Cameron_-_John_Herschel_(Metropolitan_Museum_of_Art_copy,_restored)_(cropped).jpg

Later scientists echoed Bacon’s concern. For instance, Sir John Herschel (1792–1871; Fig. 2), one of the most impressive polymaths of his time,^3^ described the state of mind required for scientific progress as follows:

But before experience itself can be used with advantage, there is one preliminary step to make, which depends wholly on ourselves: it is the absolute dismissal and clearing the mind of all prejudice, from whatever source arising, and the determination to stand and fall by the result of a direct appeal to facts in the first instance, and of strict logical deduction from them afterward. . . .

But it is unfortunately the nature of prejudices of opinion to adhere, in a certain degree, to every mind, and to some with pertinacious obstinacy, *pigris radicibus*, after all ground for their reasonable entertainment is destroyed. Against such a disposition the student of natural science must contend with all his power. Not that we are so unreasonable as to demand of him an instant and peremptory dismission of all his former opinions and judgments; all we require is, that he will hold them without bigotry, retain till he shall see reason to question them, and be ready to resign them when fairly proved untenable, and to doubt them when the weight of probability is shown to lie against them. If he refuse this, he is incapable of science. (Herschel, 1851, pp. 80–81)

**Fig. 3.**
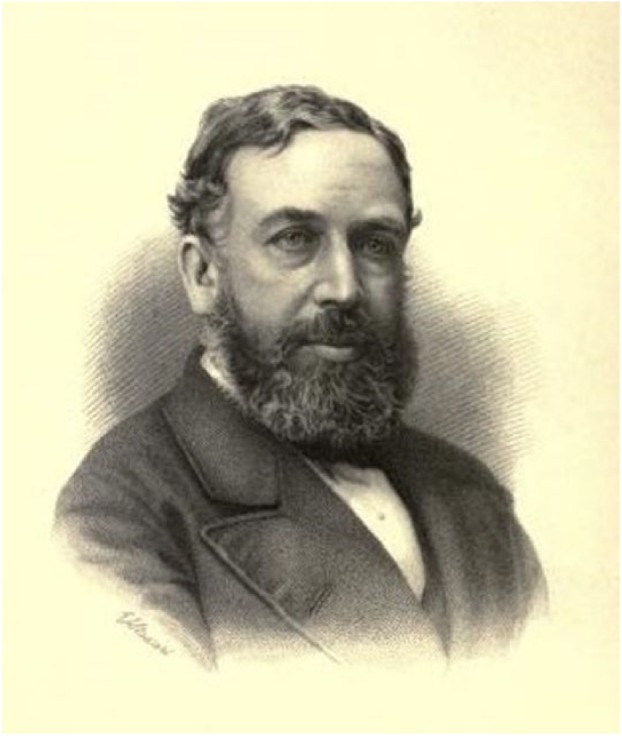
William Stanley Jevons (1835–1882). Portrait at the age of 42 by G. F. Stodart. Public-domain image available at https://en.wikipedia.org/wiki/William_Stanley_Jevons#/media/File:William_Stanley_Jevons.jpg

Another stern warning was issued by Jevons (1877/1913; Fig. 3), a highly influential economist and philosopher of science. In his book *The Principles of Science*, in the section “Mental Conditions of Correct Observation,” Jevons noted that

Every observation must in a certain sense be true, for the observing and recording of an event is in itself an event. But before we proceed to deal with the supposed meaning of the record, and draw inferences concerning the course of nature, we must take care to ascertain that the character and feelings of the observer are not to a great extent the phenomena recorded. The mind of man, as Francis Bacon said, is like an uneven mirror, and does not reflect the events of nature without distortion. We need hardly take notice of intentionally false observations, nor of mistakes arising from defective memory, deficient light, and so forth. Even where the utmost fidelity and care are used in observing and recording, tendencies to error exist, and fallacious opinions arise in consequence. It is difficult to find persons who can with perfect fairness register facts for and against their own peculiar views. Among uncultivated observers the tendency to remark favourable and forget unfavourable events is so great, that no reliance can be placed upon their supposed observations. (p. 402)

In sum, experience and experiment have shown that cognitive biases can unduly influence human reasoning and, by extension, may corrupt human research. Francis Bacon proposed to eliminate these biases by applying a mechanical inductive procedure, but by stifling creativity he paid too high a price. So how can we resolve the tension between, on the one hand, the need for creativity and discovery and, on the other hand, the need for unbiased, rigorous verification?

### Whewell’s staircase

**Fig. 4.**
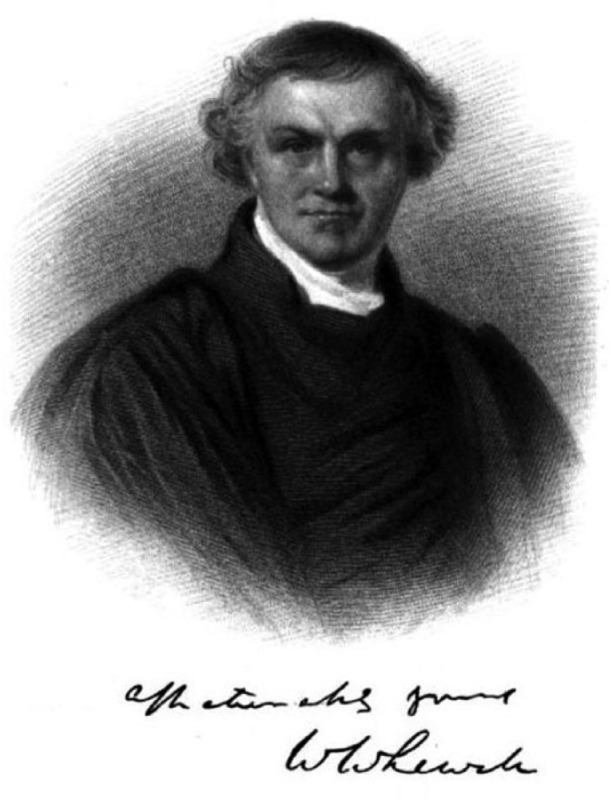
William Whewell (1794–1866). Engraving of a portrait. Public-domain image available at https://en.wikipedia.org/wiki/William_Whewell#/media/File:Whewell_William_signature.jpg

William Whewell (1794–1866; Fig. 4) was another remarkable scientist. In fact, Whewell coined the word “scientist.”^4^ Whewell proposed that the scientific process consists of two mutually reinforcing modes of reasoning: an inductive mode, which involves a creative leap, and a deductive mode, which carefully checks whether the leap was justified (see also Herschel, 1851, pp. 174–175):

The doctrine which is the hypothesis of the deductive reasoning, is the inference of the inductive process. The special facts which are the basis of the inductive inference, are the conclusion of the train of deduction. And in this manner the deduction establishes the induction. The principle which we gather from the facts is true, because the facts can be derived from it by rigorous demonstration. Induction moves upward and deduction downwards on the same stair.

But still there is a great difference in the character of their movements. Deduction descends steadily and methodically, step by step: Induction mounts by a leap which is out of the reach of method. She bounds to the top of the stair at once; and then it is the business of Deduction, by trying each step in order, to establish the solidity of her companion’s footing. (Whewell, 1840, p. 257)

Whewell’s staircase brings creativity and verification under the same umbrella. But it remains unclear how to combat Bacon’s Idols, other than by stern warnings. In other words, how do we differentiate between the inductive process and the deductive process?

### Peirce’s rules

**Fig. 5.**
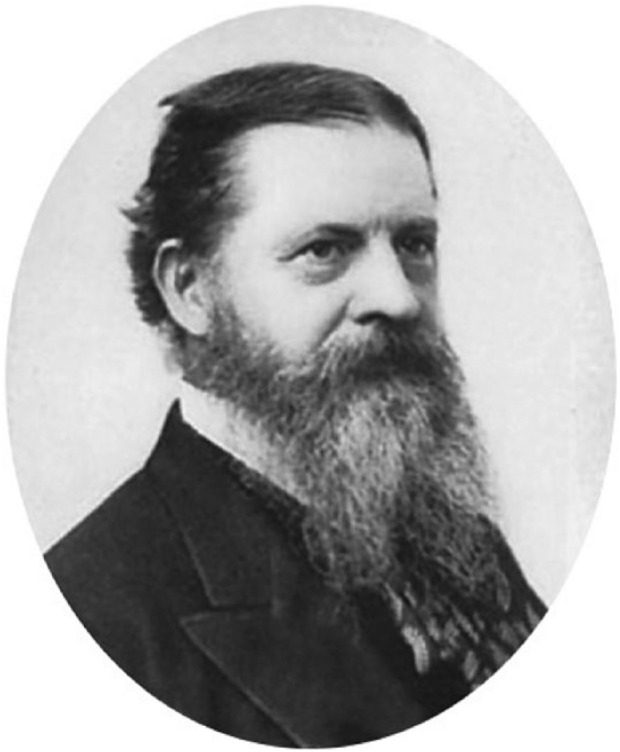
Charles Sanders Peirce (1839–1914). Public-domain image available at https://en.wikipedia.org/wiki/Charles_Sanders_Peirce#/media/File:Charles_Sanders_Peirce.jpg

C. S. Peirce (pronounced “purse”; 1839–1914; Fig. 5) was a chemist, mathematician, logician, and philosopher of science. Central among Peirce’s philosophical interests were questions concerning the scientific process and how to obtain and quantify support in favor of a hypothesis. Peirce proposed a set of three simple rules for obtaining reliable results:

In order that the process of making an hypothesis should lead to a probable result, the following rules must be followed:

1. The hypothesis should be distinctly put as a question, before making the observations which are to test its truth. In other words, we must try to see what the result of predictions from the hypothesis will be.

2. The respect in regard to which the resemblances are noted must be taken at random. We must not take a particular kind of predictions for which the hypothesis is known to be good.

3. The failures as well as the successes of the predictions must be honestly noted. The whole proceeding must be fair and unbiased. (Peirce, 1878, p. 476)

The first rule anticipates preregistration (e.g., Chambers, 2013; Wagenmakers, Wetzels, Borsboom, van der Maas, & Kievit, 2012), the second rule anticipates Popper’s idea of a severe test (e.g., Popper, 1962; see also Mayo, 1991; Mayo & Spanos, 2011), and the third rule is reminiscent of the Cicero-Bacon “temple of Neptune.”

Peirce distinguished three stages of scientific inquiry: deduction, induction, and abduction. *Abduction*, or simply “guessing,” is the creative stage in which researchers generate hypotheses, the predictions of which are later put to the test.^5^ Peirce argued that the distinct stages of inquiry be strictly separated and that legitimate inductive inference (i.e., learning from observations) requires “predesignation,” or the advance formulation of precise predictions, as specified by the first rule above.

### De Groot’s cycle

**Fig. 6.**
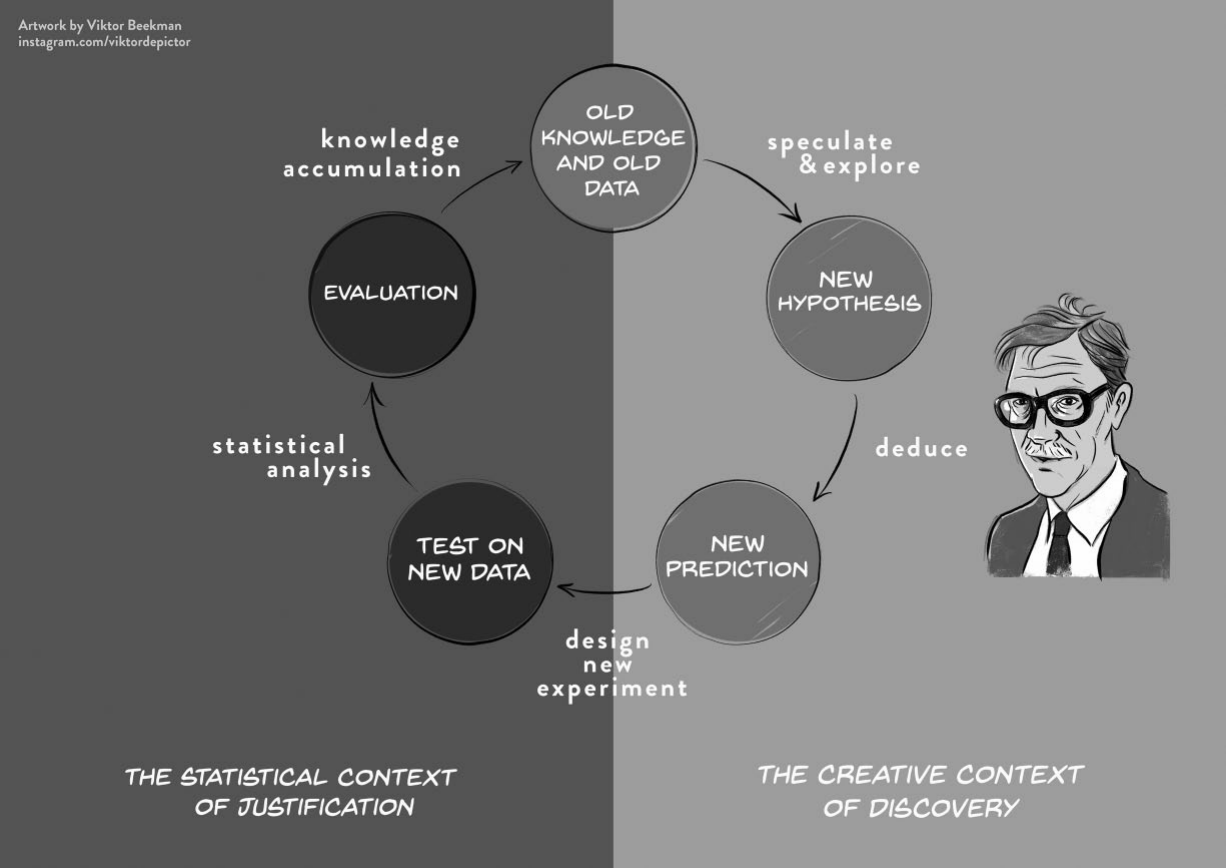
De Groot’s empirical cycle. We added the Whewell-Peirce-Reichenbach distinction between the context of discovery and the context of justification. CC-BY: Artwork by Viktor Beekman, concept by Eric-Jan Wagenmakers.

Adrianus Dingeman de Groot (1914–2006) was a Dutch methodologist whose ideas about scientific inquiry were inspired in part by Popper. Although his major work “Methodology” (de Groot, 1961/1969) fails to mention Peirce, the ideas of de Groot and Peirce were closely aligned. De Groot’s “empirical cycle,” shown here in Figure 6, provides a systematic overview of the scientific growth of knowledge. We start at the top with “old knowledge and old data.” Next we enter a Peirceian “abductive” phase of speculation and exploration. This is the phase in which the creative researcher enjoys complete freedom:

The construction of hypotheses about possible associations in reality is principally considered a “free” activity. . . . Only when this freedom is respected will room remain for the brilliant insight, for the imagination of the researcher. (de Groot, 1961, p. 38; my translation from the Dutch)

Once creative speculation has spawned a new hypothesis, this gives rise to new predictions, which can then be tested in a new experiment. The inductive evaluation of the outcome results in an updated knowledge base, after which the empirical cycle starts anew.

As did Peirce, de Groot repeatedly stressed the importance of maintaining the integrity of the empirical cycle and not allowing shortcuts, however beguiling (e.g., de Groot, 1956/2014). Specifically, to test a new prediction, one needs a fresh data set: The old data that were used to *generate* the new prediction may not be reused to *test* that prediction:

It is of the utmost importance at all times to maintain a clear distinction between exploration and hypothesis testing. The scientific significance of results will to a large extent depend on the question whether the hypotheses involved had indeed been antecedently formulated, and could therefore be tested against genuinely new materials. Alternatively, they would, entirely or in part, have to be designated as ad hoc hypotheses, which could, emphatically, not yet be tested against “new” materials. . . .

It is a serious offense against the social ethics of science to pass off an exploration as a genuine testing procedure. Unfortunately, this can be done quite easily by making it appear as if the hypotheses had already been formulated before the investigation started. Such misleading practices strike at the roots of “open” communication among scientists. (de Groot, 1961/1969, p. 52)

In other words, “You cannot find your starting hypothesis in your final results” (Goldacre, 2009, p. 221). To prevent this from occurring, de Groot (1961/1969) recommended a detailed preregistration effort:

If an investigation into certain consequences of a theory or hypothesis is to be designed as a genuine testing procedure (and not for exploration), a precise antecedent formulation must be available, which permits testable consequences to be deduced. (p. 69)

De Groot’s ideas about the empirical cycle were partly inspired by work on human problem solving. A strong chess player himself,^6^ de Groot felt it was natural that the accumulation of knowledge involved separate stages, including the creative generation of a “candidate move” followed by a critical and impartial evaluation of the consequences of that candidate move.

## Conclusion

The tension between creativity and verification lies at the heart of most theories of scientific inquiry. Francis Bacon focused mostly on the fallacies of individual human judgment and cautioned against unwarranted leaps in imagination: “The understanding must not therefore be supplied with wings, but rather hung with weights, to keep it from leaping and flying” (Bacon, 1620/1858, p. 97).

As pointed out by later scientists, creativity and verification play complementary roles in different stages of the scientific process. Early in the process, when hypotheses need to be generated from a present body of knowledge, the understanding may well be supplied with wings. But this is allowed only because in the next stages, it is hung with weights. Without verification in place, the only recourse would be to adopt a mechanical, Baconian view of creativity. Moreover, creative processes benefit from having a reliable knowledge base, and this is something that the verification process helps establish.

As a method to ensure that creativity and verification retain their rightful place in the empirical cycle, preregistration presents a conceptually straightforward solution. However, preregistration is not a panacea. For instance, in a study on randomized clinical trials, Chan, Hróbjartsson, Haahr, Gøtzsche, and Altman (2004) report that “in comparing published articles with protocols, 62% of trials had at least 1 primary outcome that was changed, introduced, or omitted. Eighty-six percent of survey responders (42/49) denied the existence of unreported outcomes despite clear evidence to the contrary” (p. 2457). This means that even with preregistration in place, researchers find it difficult to overcome their biases (see also http://compare-trials.org/). It may well be that Chambers’s Registered Report format would alleviate this issue, given that the reviewers are explicitly tasked to confirm whether the authors deviated from their protocol. Of course, without the benefit of a preregistration protocol, it would be impossible to learn that primary outcome measures have been switched altogether.

A possible limitation of preregistration is that one might encounter unforeseen data patterns (e.g., a pronounced fatigue effect) that necessitate an adjustment of the initial analysis plan. The adjusted analyses—no matter how sensible and appropriate—cannot retain the status of “confirmatory” and are automatically demoted to “exploratory.” Such a harsh penalty is fitting when the researcher has unwittingly violated the empirical cycle; however, when it is doled out for executing sound statistical judgment, this seems counterproductive to the point of being wasteful and unfair. A method that could help here is blinding (e.g., MacCoun & Perlmutter, 2015), possibly in combination with preregistration (Dutilh et al., 2017); in a blinded analysis, the analyst is initially given only part of the data—enough to create a sensible statistical model and account for unexpected data patterns but not enough to tweak the model so that it can produce the desired outcome. For instance, in the Dutilh et al. (2017) article, the analyses of interest involved various correlations with a measure of working memory capacity; the blinded analyst was given access to all data, except that the working memory scores were randomly shuffled across participants. Only after the analyst had committed to a specific analysis was the blind lifted, and the proposed analyses applied—without any change—to the unshuffled data set. Note that the analyst did alter some of the preregistered analyses, but because the analyst was blinded, the end result nevertheless retained its confirmatory status.

A final concern is that preregistration as it is currently practiced is often not sufficiently specific (Veldkamp, 2017, chap. 6). To protect against hindsight bias and confirmation bias, a proper preregistration document must indicate exactly what analyses are planned, leaving no room for doubt. But in the absence of an actual data set, this can sometimes be difficult to do in advance; consequently, the preregistration document may leave room for alternative interpretation (e.g., “Were we going to analyze the data combined or for each task separately?”; “Were we going to test the ANOVA interaction or look at the two main effects?”). A possible way to alleviate this concern is to devise the analysis plan on the basis of one or more mock data sets (e.g., Wagenmakers et al., 2016); the mock data set is generated to resemble the expected real data set and forms a concrete testing case for the preregistration plan. In addition, a mock data set makes it easier to write specific analysis code that could later be executed mechanically on the real data set.

As preregistration becomes more popular, new challenges may arise and new solutions will be developed to address these challenges. Although preregistration may have drawbacks, we do not believe that the increased focus on verification will hinder the discovery of new ideas. Creativity and verification are not competing forces in a zero-sum game; instead, they are in a symbiotic relationship in which neither could function properly in the absence of the other. It turns out that our understanding needs both wings and weights.

## Notes

1. Lest the reader be left with the impression that Francis Bacon is universally heralded as the messiah of the scientific method, here is the merciless judgment by von Liebig: “When a boy, [Bacon] studied jugglery; and his cleverest trick of all, that of deceiving the world, was quite successful. Nature, that had endowed him so richly with her best gifts, had denied him all sense for Truth” (von Liebig, 1863, p. 261).

2. “Diagoras, named the Atheist, once came to Samothrace, and a certain friend said to him, ‘You who think that the gods disregard men’s affairs, do you not remark all the votive pictures that prove how many persons have escaped the violence of the storm, and come safe to port, by dint of vows to the gods?’ ‘That is so,’ replied Diagoras; ‘it is because there are nowhere any pictures of those who have been shipwrecked and drowned at sea’” (Cicero, 45 B.C./1933, p. 375).

3. Among his varied academic exploits, Herschel named seven moons of Saturn and four moons of Uranus.

4. “What is most often remarked about Whewell is the breadth of his endeavours. In a time of increasing specialisation, Whewell appears as a vestige of an earlier era when natural philosophers dabbled in a bit of everything. He researched ocean tides (for which he won the Royal Medal), published work in the disciplines of mechanics, physics, geology, astronomy, and economics, while also finding the time to compose poetry, author a Bridgewater Treatise, translate the works of Goethe, and write sermons and theological tracts. In mathematics, Whewell introduced what is now called the Whewell equation, an equation defining the shape of a curve without reference to an arbitrarily chosen coordinate system” (“William Whewell,” 2017).

5. In a 1901 article (reprinted in Peirce, 1985), Peirce stressed the fact that abduction is “nothing but guessing” (p. 753), noting that abduction makes its start from the facts, without, at the outset, having any particular theory in view, though it is motived [*sic*] by the feeling that a theory is needed to explain the surprising facts. . . . The mode of suggestion by which, in abduction, the facts suggest the hypothesis is by *resemblance*,—the resemblance of the facts to the consequences of the hypothesis.” (pp. 752–753)

6. De Groot represented the Netherlands in the Chess Olympiads of 1937 and 1939; his translated thesis “Thought and Choice in Chess” laid the foundation for much of the later work in human problem solving (de Groot, 1946, 1965).

## References

[bibr1-1745691618771357] Bacon F. (1858a). Novum organum, in Spedding J. Ellis R. L. Heath D. D. (Eds.), The works of Francis Bacon (pp. 39–248). London, England: Longman (Original work published 1620). Retrieved from https://archive.org/details/worksfrancisbac00heatgoog

[bibr2-1745691618771357] Bacon F. (1858b). Of the dignity and advancement of learning, in Spedding J. Ellis R. L. Heath D. D. (Eds.), The works of Francis Bacon (pp. 275–498). London, England: Longman (Original work published 1605). Retrieved from https://archive.org/details/worksfrancisbac00heatgoog

[bibr3-1745691618771357] Chambers C. D. (2013). Registered reports: A new publishing initiative at Cortex. Cortex, 49, 609–610. 23347556 10.1016/j.cortex.2012.12.016

[bibr4-1745691618771357] Chambers C. D. (2017). The seven deadly sins of psychology: A manifesto for reforming the culture of scientific practice. Princeton, NJ: Princeton University Press.

[bibr5-1745691618771357] Chan A.-W. Hróbjartsson A. Haahr M. T. Gøtzsche P. C. Altman D. G. (2004). Empirical evidence for selective reporting of outcomes in randomized trials: Comparison of protocols to published articles. Journal of the American Medical Association, 291, 2457–2465. 15161896 10.1001/jama.291.20.2457

[bibr6-1745691618771357] Cicero M. T. (1933). De natura deorum [On the nature of the gods] (Rackham H., Trans.). Cambridge, MA: Harvard University Press (Original work composed ca. 45 B.C.). Retrieved from https://archive.org/details/denaturadeorumac00ciceuoft

[bibr7-1745691618771357] de Groot A. D (1946). Het denken van den schaker: Een experimenteel-psychologische studie [The thinking of the chess player: An experimental psychological study]. Amsterdam, The Netherlands: Noord-Hollandsche Uitgevers Maatschappij.

[bibr8-1745691618771357] de Groot A. D (1965). Thought and choice in chess. The Hague, The Netherlands: Mouton.

[bibr9-1745691618771357] de Groot A. D (1961). Methodologie: Grondslagen van onderzoek en denken in de gedragswetenschappen [Methodology: Foundations of inference and research in the behavioral sciences]. The Hague, The Netherlands: Mouton.

[bibr10-1745691618771357] de Groot A. D (1969). Methodology: Foundations of inference and research in the behavioral sciences (Spiekerman J. A. A., Trans.). The Hague, The Netherlands: Mouton. (Original work published 1961)

[bibr11-1745691618771357] de Groot A. D (2014). The meaning of “significance” for different types of research [translated and annotated by Eric-Jan Wagenmakers, Denny Borsboom, Josine Verhagen, Rogier Kievit, Marjan Bakker, Angelique Cramer, Dora Matzke, Don Mellenbergh, and Han L. J. van der Maas]. Acta Psychologica, 148, 188–194. (Original work published 1956) 24589374 10.1016/j.actpsy.2014.02.001

[bibr12-1745691618771357] Dutilh G. Vandekerckhove J. Ly A. Matzke D. Pedroni A. Frey R.. . . Wagenmakers E.-J. (2017). A test of the diffusion model explanation for the worst performance rule using preregistration and blinding. Attention, Perception, & Psychophysics, 79, 713–725. 10.3758/s13414-017-1304-y PMC5352774 28233280

[bibr13-1745691618771357] Fischhoff B. (1975). Hindsight is not equal to foresight: The effect of outcome knowledge on judgment under uncertainty. Journal of Experimental Psychology: Human Perception and Performance, 1, 288–299.

[bibr14-1745691618771357] Goldacre B. (2009). Bad science. London, England: Fourth Estate.

[bibr15-1745691618771357] Herschel J. F. W. (1851). Preliminary discourse on the study of natural philosophy (2nd ed.). London, England: Longman, Brown, Green, and Longmans.

[bibr16-1745691618771357] Jevons W. S. (1913). The principles of science: A treatise on logic and scientific method. London, England: Macmillan. (Original work published 1877)

[bibr17-1745691618771357] Lilienfeld S. O. (2017). *Clinical Psychological Science*: Then and now. Clinical Psychological Science, 5, 3–13.

[bibr18-1745691618771357] Lindsay D. S. (2015). Replication in psychological science. Psychological Science, 26, 1827–1832. 26553013 10.1177/0956797615616374

[bibr19-1745691618771357] MacCoun R. Perlmutter S. (2015). Hide results to seek the truth. Nature, 526, 187–189. 26450040 10.1038/526187a

[bibr20-1745691618771357] Mayo D. G. (1991). Novel evidence and severe tests. Philosophy of Science, 58, 523–552.

[bibr21-1745691618771357] Mayo D. G. Spanos A. (2011). Error statistics. In Bandyopadhyay P. S. Forster M. R. (Eds.), Handbook of the philosophy of science: Vol. 7. Philosophy of statistics (pp. 1–46). Amsterdam, The Netherlands: Elsevier.

[bibr22-1745691618771357] Nickerson R. S. (1998). Confirmation bias: A ubiquitous phenomenon in many guises. Review of General Psychology, 2, 175–220.

[bibr23-1745691618771357] Nosek B. A. Alter G. Banks G. C. Borsboom D. Bowman S. D. Breckler S. J.. . . Yarkoni T. (2015). Promoting an open research culture. Science, 348, 1422–1425. 26113702 10.1126/science.aab2374 PMC4550299

[bibr24-1745691618771357] Nosek B. A. Ebersole C. R. DeHaven A. C. Mellor D. T. (2018). The preregistration revolution. Proceedings of the National Academy of Sciences, USA, 115, 2600–2606. doi:10.1073/pnas.1708274114 PMC5856500 29531091

[bibr25-1745691618771357] Nosek B. A. Lindsay D. S. (2018 3). Preregistration becoming the norm in psychological science. APS Observer, 31 Retrieved from https://www.psychologicalscience.org/observer/preregistration-becoming-the-norm-in-psychological-science

[bibr26-1745691618771357] Pashler H. Wagenmakers E.-J. (2012). Editors’ introduction to the special section on replicability in psychological science: A crisis of confidence? Perspectives on Psychological Science, 7, 528–530. 26168108 10.1177/1745691612465253

[bibr27-1745691618771357] Peirce C. S. (1877 11). Illustrations of the logic of science: First paper—The fixation of belief. Popular Science Monthly, 12, 1–15. Retrieved from https://archive.org/details/popularsciencemo12newy

[bibr28-1745691618771357] Peirce C. S. (1878 8). Illustrations of the logic of science: Sixth paper—Deduction, induction, and hypothesis. Popular Science Monthly, 13, 470–482. Retrieved from https://archive.org/details/popularsciencemo13newy

[bibr29-1745691618771357] Peirce C. S. (1883). A theory of probable inference. In Peirce C. S. (Ed.), Studies in logic (pp. 126–181). Boston, MA: Little, Brown.

[bibr30-1745691618771357] Peirce C. S. (1985). On the logic of drawing history from ancient documents especially from testimonies [690] 1901, in Eisele C. (Ed.), Historical perspectives on Peirce’s logic of science: A history of science (pp. 705–800). Berlin, Germany: Walter de Gruyter.

[bibr31-1745691618771357] Popper K. R. (1962). Conjectures and refutations: The growth of scientific knowledge. New York, NY: Basic Books.

[bibr32-1745691618771357] Pronin E. Lin D. Y. Ross L. (2002). The bias blind spot: Perceptions of bias in self versus others. Personality and Social Psychology Bulletin, 28, 369–381.

[bibr33-1745691618771357] Spellman B. A. Gilbert E. A. Corker K. S. (2018). Open science. In Wixted J. Wagenmakers E.-J. (Eds.), Stevens’ handbook of experimental psychology and cognitive neuroscience, Volume 5: Methodology (4th ed., pp. 729–776). New York, NY: John Wiley.

[bibr34-1745691618771357] van ‘t Veer A. E. Giner-Sorolla R (2016). Pre-registration in social psychology—A discussion and suggested template. Journal of Experimental Social Psychology, 67, 2–12.

[bibr35-1745691618771357] Vazire S. (2018). Implications of the credibility revolution for productivity, creativity, and progress. Perspectives on Psychological Science, 13, 411–417. doi:10.1177/1745691618751884 29961410

[bibr36-1745691618771357] Veldkamp C. L. S. (2017). The human fallibility of scientists: Dealing with error and bias in academic research (Unpublished doctoral dissertation). University of Tilburg, The Netherlands Retrieved from https://psyarxiv.com/g8cjq/

[bibr37-1745691618771357] von Liebig J (1863, May-Oct). Lord Bacon as natural philosopher. Part II. Macmillan’s Magazine, 8, 257–267. Retrieved from https://babel.hathitrust.org/cgi/pt?id=uc1.$b676993;view=1up;seq=249

[bibr38-1745691618771357] Wagenmakers E.-J. Beek T. Dijkhoff L. Gronau Q. F. Acosta A. Adams R.. . . Zwaan R. A. (2016). Registered Replication Report: Strack, Martin, & Stepper (1988). Perspectives on Psychological Science, 11, 917–928. 27784749 10.1177/1745691616674458

[bibr39-1745691618771357] Wagenmakers E.-J. Wetzels R. Borsboom D. van der Maas H. L. J. Kievit R. A. (2012). An agenda for purely confirmatory research. Perspectives on Psychological Science, 7, 627–633. 10.1177/1745691612463078 26168122

[bibr40-1745691618771357] Whewell W. (1840). The philosophy of the inductive sciences, founded upon their history (Vol. 2). London, England: John W. Parker Retrieved from https://archive.org/details/philosophyofindu01whewrich.

[bibr41-1745691618771357] William Whewell. (2017). In Wikipedia. Retrieved 1 5, 2017, from https://en.wikipedia.org/w/index.php?title=William_Whewell&oldid=758490609

